# Correction: Mackala et al. Evaluation of the Pre-Planned and Non-Planed Agility Performance: Comparison between Individual and Team Sports. *Int. J. Environ. Res. Public Health* 2020, *17*, 975

**DOI:** 10.3390/ijerph20126174

**Published:** 2023-06-19

**Authors:** Krzysztof Mackala, Janez Vodičar, Milan Žvan, Jožef Križaj, Jacek Stodolka, Samo Rauter, Jožef Šimenko, Milan Čoh

**Affiliations:** 1 Department of Track and Field, University School of Physical Education, Wroclaw, Ul. Paderewskiego 35, 51-612 Wrocław, Poland; jacek.stodolka@awf.wroc.pl; 2 Faculty of Sport, University of Ljubljana, Gortanova ul. 22, 1000 Ljubljana, Slovenia; janez.vodicar@fsp.uni-lj.si (J.V.); milan.zvan@fsp.uni-lj.si (M.Ž.); jozef.krizaj@fsp.uni-lj.si (J.K.); samo.rauter@fsp.uni-lj.si (S.R.); jozef.simenko@fsp.uni-lj.si (J.Š.); milan.coh@fsp.uni-lj.si (M.Č.); 3 School of Life and Medical Sciences, University of Hertfordshire, Hatfield AL10 9EU, UK

## Addition of an Author

**Jožef Šimenko** was not included as an author in the original publication [1]. And this author’s affiliation: “School of Life and Medical Sciences, University of Hert-fordshire, Hatfield AL10 9EU, UK”, also need to add. The corrected Author Contributions statement appears here. The authors state that the scientific conclusions are unaffected. This correction was approved by the Academic Editor. The original publication has also been updated.
